# Supplementary material for: Patterns of Intron Gain and Loss in Fungi
Source: PLoS Biol. 2004 Nov 30;2(12):e422. doi: 10.1371/journal.pbio.0020422 (PMC532390; doi:10.1371/journal.pbio.0020422)
Supplement: Table S1 — Also available at http://genes.mit.edu/NielsenEtAl/. (4.3 MB ZIP). [file pbio.0020422.st001.zip › NielsenEtAl/html/933.html]

AN0428.1.NCU00931.1.MG00161.1.FG00875.1


```
 CLUSTAL W (1.82) Multiple Sequence Alignments - Introns Inserted


Sequence 1: NCU00931.1	594 aa
Sequence 2: FG00875.1	581 aa
Sequence 3: AN0428.1	527 aa
Sequence 4: MG00161.1	638 aa
Alignment Length: 705 aa
Number Identitical Residues: 122 aa
Alignment Score (without introns) 6932


MG00161.1 	MFSLGRSSLRCAHRAGLPRPRLASEV~VVQNRTLPRCYSVSRDPSE~LKGRREPAASEPS
NCU00931.1	------------------------MI0FLRPYAWKWKPVGARNTAY~LRFYHLARQ-HEL
FG00875.1 	--------------------------~------MEHTKCDKR---A0RRTELWAEQ-EEE
AN0428.1  	--------------------------~----MRIRSCRVACRSFVP~GRWYSKATSSKEG
          	                                         *.     :    .  :.  

MG00161.1 	NFLSSVKDAKPNQTGQATTQVDAVKPSWTVESKTTTKIKNIADMPLSKLFDVCPSDSNSG
NCU00931.1	IQSNALKYPRIRHEGGAPMRIPTFR----------------------------------E
FG00875.1 	DPLFTETHPRLVNR-ATRMSVPEFY----------------------------------E
AN0428.1  	SLKQTLSKEALSKKQAFVTRLAQVT----------------------------------E
          	    : .     :       :  .                                    

MG00161.1 	RMRVVRGIEDIDEPLTAKGRIMNIRSHGS-LIFLDIAGDYETL~QVA~IS-YAFLKEKSP
NCU00931.1	KFKDVEMGKHADEEVIIHGRVQSVRRAGNKLMFLDLKGEFEHV~QGL~CNFNKLQVTG--
FG00875.1 	TFDSCLD---DSNDITVYGRIRSKRVVGKNLIFIDIVNEFTRL~QVM~VNRSKCMVDQED
AN0428.1  	ATSDPYPRFETPANPFAISEFIHRYSYLANKEFVDFDQVTVTG1RVH1PLQFESPGTGRG
          	                  ...        .  *:*:        :               

MG00161.1 	AEMEGVSLRDFKAKGAQLQ~RGD-WVV~AAGHMEADRRDGLRMRAHNFPSIRAKALNIPP
NCU00931.1	--VELGEFKALAKL---LN~RGD-IVS1ITGRATKTPTGELSIEATRLPELLTPALAPLP
FG00875.1 	RRLKFGMFRSLIEVGDHIY1AYS-LAA~ITGIPTRTKAGELTIEAKALPELLSPTMEQIP
AN0428.1  	LKADIQEILSYDSPRRCLL2LNQNATA~ITGKPHRTERGELSIVATDLPQLLSPCLHDIP
          	   .   :         :    .. .   :*       . * : *  :*.: :  :   *

MG00161.1 	LSVIDDGLMSQSRHLDLMLNKVSVDALRARARIIQTVRRTLDDRGYVEVQTPLLAGSAGG
NCU00931.1	FKLEDEEVRIQHRHIDMLVNRKTIDTLRLRSHLIKYLRDFFHERGFLEFQTPILAENASG
FG00875.1 	EKLTDPKTRMQERHVDMLVNREAIDVLRLRAEITKYMRDYFHSKRFLEFQTPILAQNAGG
AN0428.1  	LDAHGHENSPYPRHVQMLGDPATVKVIKSRSLITQHIRQFLLDKSFMEVSTPILNGIPGG
          	 .  .       **:::: :  ::..:: *: : : :*  : .: ::*..**:*   ..*

MG00161.1 	ATARPFATTATEFGSSQHLALRVAPELWLKRLVMGGMDRVYEVGPCFRNEG~VDATHNPE
NCU00931.1	ATARPFTVTASETSVNKNLAMRIAPELWLKRLVVGGVDKVFELGPAFRNEG~IDQTHNPE
FG00875.1 	AVARPFVTRATEFKD-KDLALRIAPELWLKRLVIGGVDKVFEIGPSFRNEG~VDATHNPE
AN0428.1  	ASARPFYTSATEFPD-RQLALRIAPELWLKRLVVGGFDRVFEIGPSFRNEG1LDKTHNPE
          	* **** . *:*    :.**:*:**********:**.*:*:*:**.***** :* *****

MG00161.1 	FTMCEFYEAWADLDHLMLMTERLMQLISSDLKAASKDAEAVGRRPWLLPDMDRIASTFCE
NCU00931.1	FSMCEFYNAYANLDDLITLTEELICGAASHCRDLIATKLDS------LPAIDMSQYQRPF
FG00875.1 	FTMCEFYSAYTNLEDLIKETEEILCGLAQHSQELISTELTA------LPPIDMSRFVRPF
AN0428.1  	FTTCEFYQAYSNLEELMNTTEELLCGLAQVFIAEFNKRKE--------PPPTAIDFTIPF
          	*: ****.*:::*:.*:  **.::   :.                   *           

MG00161.1 	EGTTPKVHFITRLEQALGFKLPDLESTTALAELIMKLRERGEDADILAAADYPEQGDAAT
NCU00931.1	Q----QLEFIPALESALGFKLPDLSASSSREDLLALLKQHKIEVPGLNKPDI--------
FG00875.1 	K----RVEFVPALQEALGLRLPKLSSPDALPEVLAILKLSGIEIPGEVPS----------
AN0428.1  	H----RIDFITGIELGIDRQLPDLTAPDSLEQVTQIFTDLKLQLP--EHP----------
          	.    ::.*:. :: .:. :**.* :. :  ::   :     :      .          

MG00161.1 	TTAPMTLPKLLDDLGSKYVEKCSTVGPHRAFFVTHHPACMAPLARSGVCPITKQKVALRA
NCU00931.1	-----TLAKLLDNLASEFLEPYSLTKP---LFITHHPVCMSPLSKSFVCPKTGQHVSART
FG00875.1 	-----SLAKLLDRLAAIYLEPMSFTEP---VLIMNHPACMSPLAKSFVCPETLQLVSARA
AN0428.1  	-----TLPRLLDELCSIYVEP-QCVQP---TFIINPPECLSPLSKSFVHPDNNQRVAARA
          	     :*.:*** * : ::*  . . *    :: : * *::**::* * * . * *: *:

MG00161.1 	EFFVNGVELANMYEEEFEADEQERKFNAAAR-----------------------------
NCU00931.1	ELFINGKELANMYEEENDPASQRQKFVDQLNAAKR-------EASLAPG-----------
FG00875.1 	ELFIGGRELANMYEEENDPEAQRRKLFEHRHLVNRDDGGIDIEIPKPPEGQDVIQDPQDE
AN0428.1  	ELFIEGKEIANMYEEENSPWEQRRKFEDQLK-----------------------------
          	*:*: * *:******* ..  *.:*:    .                             

MG00161.1 	-LRRDAVGLNSDEASYVLAMRGGMPPTGGWGCGIDRLAMLFTGKPRIRDVLTFGNLRNVV
NCU00931.1	VYNDEGESAMEVDESYVQALESGLPPTGGWGCGIERMVMLFSGTKRISDCLSFGNLRHVT
FG00875.1 	PFEDKDGDAAPLDQSFVKALDYGLPPTGGWGCGIERMVMLFSGASRISDCLSFGTIRNVV
AN0428.1  	-YSKDANEPGEIDEEYLRALEWGLPPTGGWGCGIDRLVMLFTKVKKIAEVLPFGNLRHVT
          	    .       : .:: *:  *:**********:*:.***:   :* : *.**.:*:*.

MG00161.1 	HATRLPGPWNAAAKRFRL----------------------------------
NCU00931.1	NVPGAVRTGEDEEKAREMMDEVEKKGARERKREKGKEKEKEKEDMGVSDGEA
FG00875.1 	GLS------ATEENKKVSSDEQKKSDEEEKK-------------TAIEEGL-
AN0428.1  	RR--------------------------------------------------
          	
```
